# Amygdalin promotes the activity of T cells to suppress the progression of HBV-related hepatocellular carcinoma via the JAK2/STAT3 signaling pathway

**DOI:** 10.1186/s12879-020-05713-0

**Published:** 2021-01-12

**Authors:** Ruoyu Wang, Dong Zhang, Kewei Sun, Jianping Peng, Wenfang Zhu, Sihan Yin, Dan Tang, Yunan Wu

**Affiliations:** 1 Department of Hepatology, The First Hospital of Hunan University of Chinese Medicine, Changsha, 410007 Hunan China; 2 Department of Hepatology, Guangdong Hospital of Traditional Chinese Medicine in Zhuhai, Zhuhai, 519015 Guangdong China

**Keywords:** Hepatitis B virus (HBV), Hepatocellular carcinoma (HCC), Amygdalin, T cell, The JAK2/STAT3 signaling

## Abstract

**Background:**

Hepatitis B virus (HBV) infection is a high-risk factor of hepatocellular carcinoma (HCC). Cellular immune responses are essential for HCC development, and the CD4+ and CD8+ T subtypes are identified as the primary anti-tumor immune cells. In the study, we investigated the effect and mechanism of amygdalin in the cellular immune response in HBV-related HCC and HCC progression.

**Methods:**

The cell proliferation was examined by MTT analysis. Cells metastasis ability was detected by Invasion and migration assays. Quantification of apoptotic cells was performed with Flow cytometer assay. The protein levels of p-STAT3, STAT3, p-JAK2, JAK2, caspase-3, cleaved caspase-3 were detected by performing immunoblotting assays.

**Results:**

We demonstrate that amygdalin treatment could rescue the HBV-T cell viability and IFN-γ and TNF-αproduction. In HBV-T cells, the MFI levels of CD8^+^ are lower than that in NC-T cells. Moreover, the phosphorylation levels of STAT3 and JAK2 are higher in HBV-T cells, compared to those in NC-T cells, and then reduced by amygdalin treatment. Co-culture with HBV-T cells could reduce IFN-γ and TNF-α, production while increase IL-6 and IL-10 production in HepG2.2.15 cells; these alterations could be partially reversed by amygdalin pretreatment. Finally, co-culture with HBV-T cells significantly promoted the cell viability, inhibited the apoptosis, and promoted the migration of HepG2.2.15 cells, and these alterations could be partially reversed by amygdalin treatment.

**Conclusion:**

Our findings provide a rationale for further studies on the functions and mechanism of amygdalin inhibiting HBV-related HCC cell proliferation, invasion, and migration via T cell-mediated tumor immunity.

**Supplementary Information:**

The online version contains supplementary material available at 10.1186/s12879-020-05713-0.

## Background

As one of the most common types of cancer in Asia, hepatocellular carcinoma (HCC) can be caused and affected by a variety of risk factors, including hepatitis B virus (HBV) infections [1–3]. Higher levels of serum HBV DNA can be directly related to a higher risk of hepatocarcinogenesis [4].

Cellular immune responses are essential for the monitoring of malignant tumors and the control of HCC development; CD4+ and CD8+ T subtypes are identified as the primary anti-tumor immune cells [5]. The cytotoxicity mediated by HBV-specific CD8+ T subtype is not only strong and effective, but also critical for controlling HBV infection and affecting cancer progression [6]. CD4+ T subtype-mediated cytotoxicity, which is essential for virus control and anti-tumor immunity [7, 8], has attracted increasing attention in recent years. However, in hepatocellular carcinoma, the number of circulating and tumor-infiltrating T cells is increased at early stages while reduced at later stages [9].

Cytokines, growth factors, and oncogenes can induce signal transduction mediated by STAT transcription factors. Among them, the signal transducer and activator of transcription 3 (STAT3) are one of the most essential members [10]. Phosphorylated STAT3 (p-STAT3) leads to tumor progression in a variety of cancers, such as lung cancer [11], breast cancer [12], prostate cancer [13], and melanoma [14]. The tumorigenesis, invasion, and metastasis of hepatocellular carcinoma are also related to STAT3 activation [15, 16]. Interestingly, increased p-STAT3 expression in the CD4+ and CD8+ T cells in peripheral blood of patients with hepatocellular carcinoma can lead to aberrant immunological surveillance, thus promoting the development of hepatocellular carcinoma [17].

Amygdalin was one of the most popular, non-conventional, anti-cancer treatments in the 1970s when over 70, 000 cancer patients were treated with amygdalin [18]. During the past decades, amygdalin is well known for its antitumor [19–23] and anti-inflammatory activities [24–27]. Amygdalin attenuates acute liver injury induced by D-galactosamine and lipopolysaccharide by regulating the NLRP3, NF-κB, and Nrf2/NQO1 signaling pathways [24]. Via inhibiting lipopolysaccharide-inducible TNF-α and IL-1β mRNA expression, Amygdalin could suppress carrageenan-induced rat arthritis [25]. Amygdalin significantly reduced LPS-induced inflammatory cell infiltration and the production of TNF-α, IL-1β, and IL-6 in the bronchoalveolar lavage fluid (BALF) in LPS-induced acute lung injury model in murine [26]. However, the effect and mechanism of amygdalin in the cellular immune response in HBV-related HCC and HCC progression remain unclear.

Herein, we examined how amygdalin affected the proliferation of normal T cell (NC-T), and HBV-related HCC T cell (HBV-T) and the production of cytokines were determined. STAT3 and JAK2 phosphorylation in NC-T and HBV-T cells were detected with or without amygdalin treatment. Finally, we co-cultured HepG2.2.15 cells with T cells (directly/indirectly) and examined the production of cytokines, cell proliferation, apoptosis, migration, and invasion. In summary, we revealed the cellular functions of amygdalin on HBV-related HCC cells and provided a solid experimental basis for understanding the underlying mechanism.

## Methods

### Isolation and purification of human peripheral blood T lymphocytes

Fifty milliliters of peripheral blood was drawn from healthy volunteers (*n* = 10, F/M is 4/6, age is 55.6 ± 10.14 year) or patients diagnosed with HBV-related HCC (*n* = 10, F/M is 3/7, age is 59.3 ± 11.00 year). The general characteristics of voluneers and patients were shown in Table S1. The patients with HCV, HIV-coinfection, diabetes, chronic kidney diseases or autoimmune disorders were excluded from the study. PBMCs were harvested by density gradient centrifugation, washed 3 times with phosphate-buffered saline (PBS), and cultured in RPMI1640 medium at 37 °C in 5% CO_2_ for 2 h. After that, the medium was changed to remove macrophages. PBMCs were routinely cultured in RPMI1640 medium containing 10 IU/ml rhIL-2 for 4 weeks, incubated with fluorescently labeled antibody to label PBMCs, and then maintained at a low dose of rhIL-2. Total T lymphocyte numbers and T cell subpopulations were detected by Flow cytometry analysis. T lymphocytes were identified with surface markers, CD3, CD4, and CD8. CD3 represents the total number of T cells, and CD4 and CD8 represent T cell subsets. Purified T lymphocytes are collected according to the analysis results of flow cytometry.

HepG2.2.15 cells, which are capable of steady secreting HBsAg, HBeAg and intact Dane particles to the culture supernatant, were obtained from SHANGHAI CAFA BIOLOGICAL TECHNOLOGY CO., LTD and cultured in DMEM medium supplemented with 10% FBS.

### Co-culture experiments

For the co-culture experiments with T cells, HepG2.2.15 cells were seeded in 96 or 24-well flat-bottom plates at a density of 5 × 10^4^ cells/ml. After co-cultured with T cells (5 × 10^5^ cells/ml) for 24, 48 or 72 h, T cells were removed, and HepG2.2.15 cells viability, apoptosis and migration ability was assessed.

### Amygdalin treatment

Amygdalin was purchased from Macklin (Shanghai, China). T cells were treated with 5, 10, 15 or 20 μg/ml amygdalin for 24, 48 and 72 h or treated with 10 μg/ml amygdalin for 48 h, T cells were collected for further experiments.

### MTT assay

The cell proliferation was examined by MTT analysis. For T cell proliferation, T cells were stimulated with 5 μg/ml concanavalin A (Con A, Sigma-Aldrich, USA), 1 × 10^4^ cells/well T cells were seeded into 96 well plates treated with or without amygdalin for 24, 48 and 72 h. For HepG2.2.15 cells proliferation, 5 × 10^3^ cells/well HepG2.2.15 cells were co-cultured with 5 × 10^4^ cells/well T cells into 96 well plates for 24, 48 and 72 h, then removed the T cells. Twenty microliters MTT (at a concentration of 5 mg/ml; Sigma-Aldrich) was added into the 96-well plates and incubated for 4 h in a humidified incubator. After that, the supernatant was discarded and 200 μl DMSO was added to dissolve the formazan. OD_490 nm_ value was measured. The viability of the non-treated cells (control) was defined as 100%.

### Real-time PCR

Total RNA was extracted from targeted cells using Trizol reagent (Invitrogen, CA, USA), and the expression of mRNA was determined using PCR-based analyses following the methods described previously [28]. SYBR green PCR Master Mix (Qiagen, German) was used. GAPDH was employed as endogenous controls for mRNA expression determination, respectively. The data were processed using a 2^-ΔΔCT^ method. The primer sequence were listed in Table S2.

### Immunoblotting analysis

The protein levels of p-STAT3, STAT3, p-JAK2, and JAK2 were detected by performing immunoblotting assays. Target cells were lysed in RIPA buffer with 1% PMSF; proteins were loaded onto an SDS-PAGE minigel and transferred onto a PVDF membrane. The blots were probed with anti-p-STAT3 (dilution 1:2000, ab76315, Abcam, Cambridge, MA, USA), anti-STAT3 (dilution 1:2000, ab119352, Abcam), anti-p-JAK2 (dilution 1:2000, ab32101, Abcam), anti-JAK2 (dilution 1:2000,ab108596, Abcam), anti-procaspase3 (dilution 1:1000, ab32150, Abcam), anti-cleaved caspase-3 (dilution 1:1000, ab32042, Abcam) and anti-GAPDH (dilution 1:5000, ab8245, Abcam) at 4 °C overnight, subsequently incubated with the HRP-conjugated secondary antibody (15,000, Santa Cruz, USA). ECL Substrates was used to visualize signals (Millipore, MA, USA). GAPDH was used as an endogenous protein for normalization.

### Cytokines production determined by ELISA

T cells culture medium was collected for ELISA assay using ELISA kits specific for IFN-γ, TNF-α, IL-6, and IL-10 according to the manufacturer’s instructions (R&D systems, USA). First, 100 μl of Assay Diluent was added to each well. One hundred microliters serially-diluted standard samples or supernatant samples were added into the microplate and were incubated at room temperature for 2 h. After aspirating each well and washing, 200 μl of Conjugate solution to each well and incubated at room temperature for 2 h. After aspirating each well and washing, each well was added with 200 μl substrate solution at dark for 30 min; Finally, each well was added with 50 μl stop solution and the optical density was assayed immediately at 540 nm with a spectrophotometer (Bio-Rad Laboratories).

### Invasion and migration assays

HepG2.2.15 cells were co-cultured with T cells and plated on the top side of Transwell filter coated with (for invasion analysis) or without Matrigel (BD, New York, USA) (for migration analysis) in the top chamber. Cells were suspended in medium without serum and medium supplemented with serum were filled in the bottom chamber. The cells were incubated at 37 °C for 48 h. The noninvasive or non-migratoryion cells in the top chambers were removed with cotton swabs. The invaded cells or migrated cells on the lower membrane surface were fixed in 100% methanol for 10 min, air-dried, then stained staining with 0.1% crystal violet solution, and counted under a microscope.

### Flow cytometer assay

After co-culture with NC-T cells, HBV-T cells or amygdalin treated HBV-T cells for 48 h, the HepG2.2.15 were harvested for apoptosis analysis. Quantification of apoptotic cells was performed with Annexin V-FITC apoptosis detection kit (Keygen, China). Briefly, the HepG2.2.15 cell samples were harvested with 0.25% trypsin without EDTA after 48 h of infection and then washed twice with ice-cold PBS and re-suspended in 500 μl binding buffer. Then cells were incubated with 5 μl Annexin V-FITC specific antibodies and 5 μl propidium iodide (PI) then incubated for 15–20 min in the dark and detected by BD Accuri C6 flow cytometer (BD, USA) with an excitation wavelength of Ex = 488 nm and an emission wavelength of Em = 530 nm.

For T cell surface markers detection, Fixed T cells were permeabilized by adding 1 ml of cold Phosflow Perm Buffer III (BD Biosciences) and incubating for 30 min on ice. Samples were then washed and incubated with the mouse anti-human antibodies (BD Biosciences) anti-CD3-PE-Cy5, −CD4-FITC, −CD8-FITC, and p-STAT3-PE at room temperature for 1 h. Then, the cells were washed and collected for flow cytometry analysis on a BD Accuri C6 flow cytometer (BD, USA). Each experiment was repeated three times in triplicate.

### Statistical analyses

Data were expressed as means ± SD of at least three independent experiments. A one-way ANOVA was used for the comparison of the differences among more three groups. The level of significance was based on the probability of *P < 0.05, **P < 0.01.

## Results

### Effect of amygdalin on normal (NC-T) and HBV-related HCC T cells (HBV-T)

**Fig. 1:**
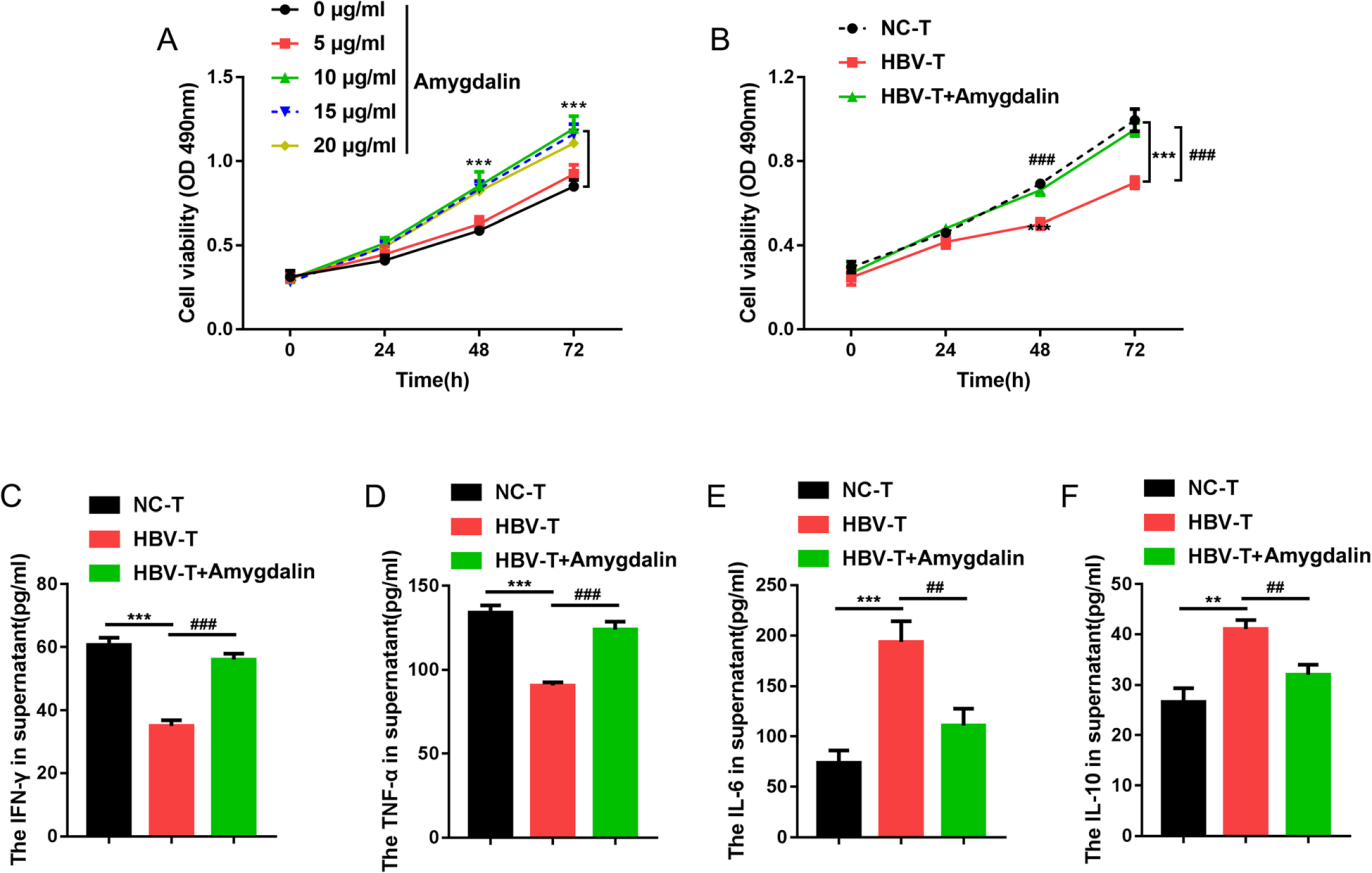
Effect of amygdalin on normal (NC-T) and HBV-related HCC T cells (HBV-T). **a** T cells isolated from the peripheral blood from healthy donors (NC-T) were stimulated with 5 μg/ml Con A and treated with a series of doses of amygdalin (0, 5, 10, 15, and 20 μg/ml) and examined for cell viability using MTT assay. **b** The cell viability of NC-T and T cells isolated from the peripheral blood from HBV-related HCC patients (HBV-T) was examined by MTT assay with or without amygdalin treatment (10 μg/ml). **c**-**f** The production of IFN-γ, TNF-α, IL-6, and IL-10 in NC-T and HBV-T cells was determined by ELISA with or without amygdalin treatment (10 μg/ml). *n* = 5, *p < 0.05, **p < 0.01, ***p < 0.001, compared to NC-T group, #*p* < 0.05, ##*p* < 0.01, ###*p* < 0.001, compared to HBV-T group

First, the effects of amygdalin on T cell growth and cytokine production were determined to investigate its role in HBV-related HCC. The cell viability of NC-T cells was examined upon different doses of amygdalin (0, 5, 10, 15, and 20 μg/ml) to select a proper treating dose. As shown in Fig. 1a, 10, 15, and 20 μg/ml of amygdalin treatment remarkably increased the cell viability of NC-T cells. Therefore, the minimum effective dose (10 μg/ml) was chosen for further experiments. Next, we examined NC-T and HBV-T cell growth with or without 10 μg/ml amygdalin treatment. Compared to the cell viability of NC-T cells, HBV-T cell viability was significantly inhibited; while amygdalin treatment significantly rescued the cell viability of HBV-T cell, compared to that of HBV-T cell without amygdalin treatment (Fig. 1b). As for the cytokines production, IFN-γ and TNF-α levels remarkably decreased while IL-6 and IL-10 level increased in HBV-T cells, compared to those in NC-T cells; amygdalin treatment significantly rescued IFN-γand TNF-α levels while reduced IL-6 and IL-10 levels, in comparison with those in HBV-T cells without amygdalin treatment (Fig. 1c-f).

### Effect of amygdalin on the activation of T cells and the phosphorylation of STAT3 and JAK2 in T cells

**Fig. 2:**
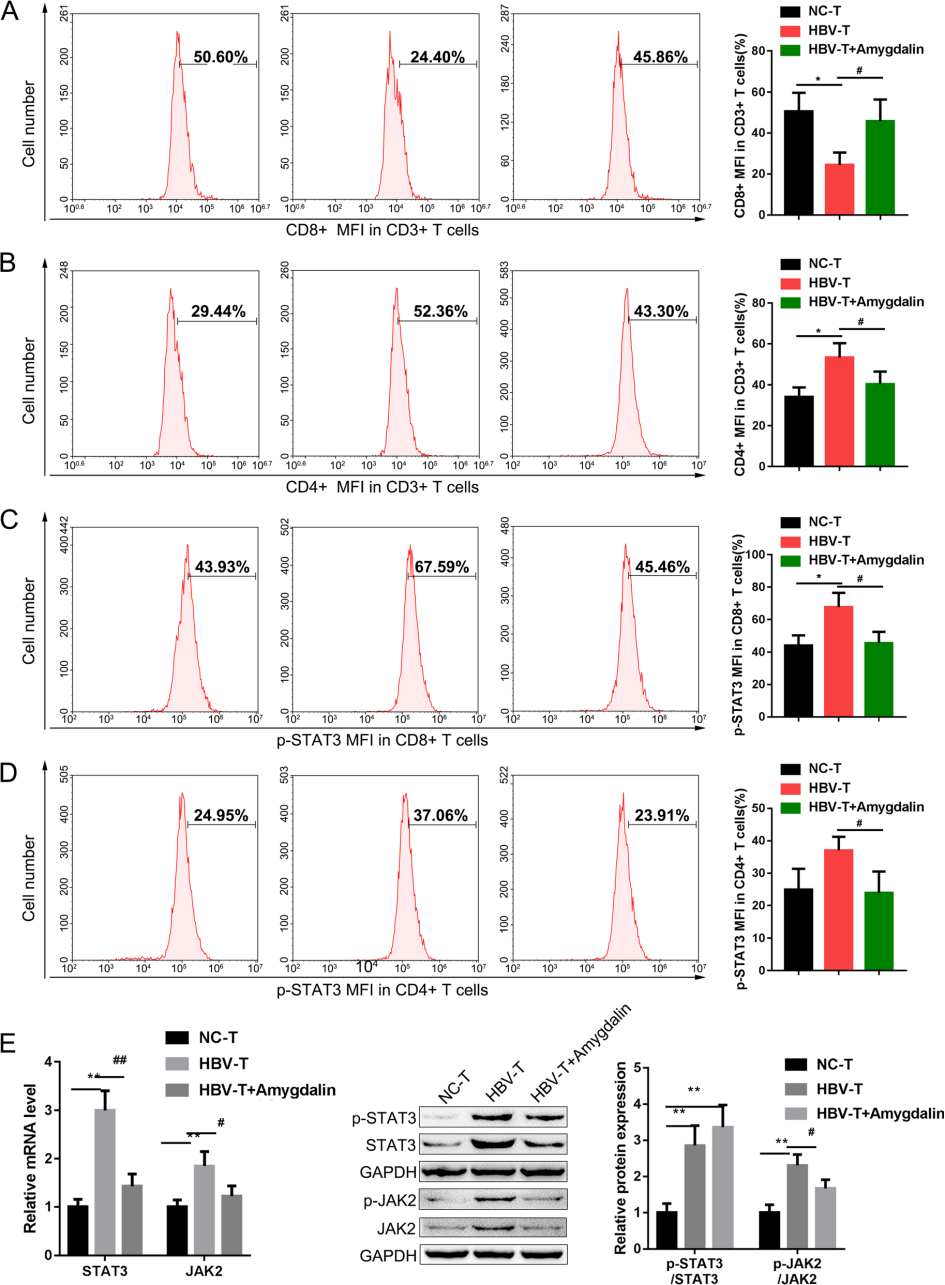
Effect of amygdalin on T cell activation and the phosphorylation of STAT3 and JAK2 in T cells. **a**, **b** The mean fluorescence intensity (MFI) of CD4^+^ and CD8^+^ T cells in total CD3^+^ T cells with or without amygdalin treatment determine by Flow cytometry analysis. *n* = 3. **c**, **d** The average mean fluorescence intensity (MFI) of p-STAT3 in CD4^+^ and CD8^+^ NC-T and HBV-T cells with or without amygdalin treatment determine by Flow cytometry analysis, *n* = 3. **e** The mRNA levels of STAT3 and JAK2 in NC-T and HBV-T cells with or without amygdalin treatment determined by real-time PCR, *n* = 3. **f** The protein levels of p-STAT3, STAT3, p-JAK2, and JAK2 in NC-T and HBV-T cells with or without amygdalin treatment determine by Immunoblotting, *n* = 3. *p < 0.05, **p < 0.01, ***p < 0.001, compared to NC-T group, #*p* < 0.05, ##*p* < 0.01, compared to HBV-T group

It has been revealed that amygdalin could rescue the cell viability of HBV-T cells, next, we investigate whether amygdalin could activate T cells via examining the ratio of CD4^+^ and CD8^+^/total T cells using Flow cytometry. According to Fig. 2a, the rate of CD8^+^ /HBV-T cells was significantly lower than that in NC-T cells; however, amygdalin treatment rescued the proportion of CD8^+^ /HBV-T cells. In contrast, the ratio of CD4^+^ /HBV-T cells was significantly higher than that in NC-T cells; amygdalin treatment reversed the proportion of CD4^+^ /HBV-T cells. These findings indicate amygdalin treatment could activate T cells in HBV-related HCC.

As we have mentioned, being increased in the CD4^+^ and CD8^+^ subtypes in peripheral blood of patients with hepatocellular carcinoma, the p-STAT3 expression can lead to aberrant immunological surveillance, thus promoting the development of hepatocellular carcinoma [17]. Here, the p-STAT3 and p-JAK2 levels in CD4^+^ and CD8^+^ NC-T and HBV-T cells were measured using Flow cytometry with or without amygdalin treatment. The results have revealed that p-STAT3 and p-JAK2 expression in CD8^+^ (Fig. 2c) and CD4^+^ subtypes (Fig. 2d) in HBV-T cells was higher than that in NC-T cells. Consistently, amygdalin treatment reduced the phosphorylation levels of STAT3 and JAK2 in CD8^+^ and CD4^+^ HBV-T cells (Fig. 2c-d), compared to those in HBV-T cells without amygdalin treatment. RT-PCR and Immunoblotting analyses reveal similar results, the mRNA levels of STAT3 and JAK2 was increased in in HBV-T cells while was reduced by amygdalin treatment. The protein ratio of p-STAT3/STAT3 and p-JAK2/JAK2 was increased in HBV-T cells while was reduced by amygdalin treatment (Fig. 2e and f). Therefore, amygdalin treatment could increase T cells activity in HBV-related HCC.

### Coculture with T cells affects the cytokine production in HepG2.2.15 cells

**Fig. 3:**
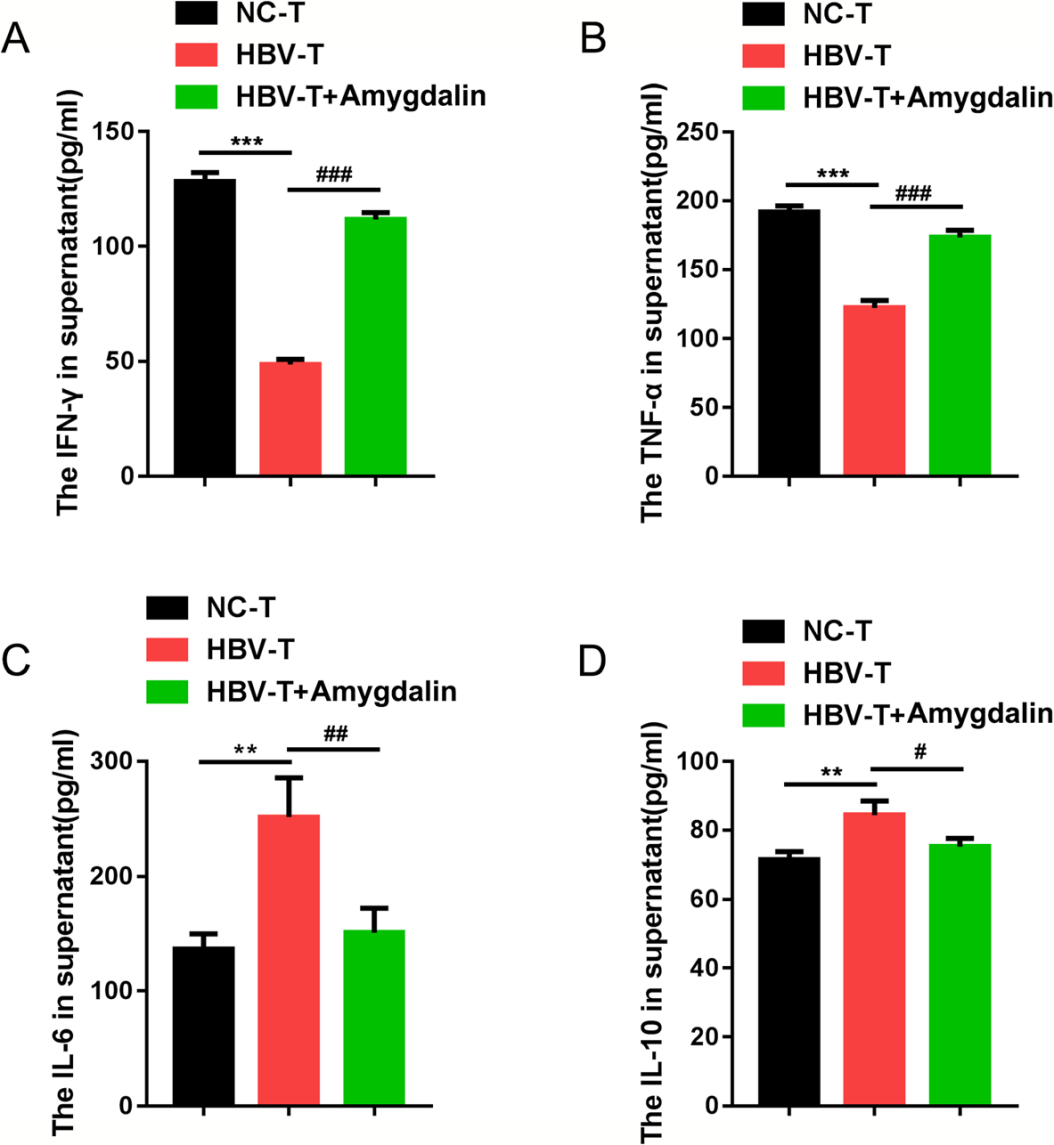
Co-culture with T cells affects the cytokine production in HepG2.2.15 cells. **a**-**d** HepG2.2.15 cells were co-cultured with NC-T, HBV-T cells or amygdalin treated HBV-T cells and examined for the production of IFN-γ, TNF-α, IL-6, and IL-10 using ELISA with or without amygdalin treatment, *n* = 5. *p < 0.05, **p < 0.01, compared to NC-T group, #*p* < 0.05, ###*p* < 0.001, compared to HBV-T group

Next, we co-cultured HCC cell line, HepG2.2.15 cell, with NC-T or HBV-T cells in the presence or absence of amygdalin therapy to investigate the effects of amygdalin on cytokine production. As shown in Fig. 3, without amygdalin treatment, co-culture with HBV-T cells significantly reduced IFN-γ and TNF-α production while increased IL-6 and IL-10 production, compared to NC-T co-culture group. Consistent with the effect on T cell viability and activity, amygdalin treatment rescued IFN-γ and TNF-αproduction while reduced IL-6 and IL-10 production in HBV-T cell co-cultured HepG2.2.15 cells. These findings indicate that amygdalin could act on HepG2.2.15 cells by affecting HBV-T cells.

### Effects of amygdalin on HepG2.2.15 cell viability, apoptosis, invasion, and migration

**Fig. 4:**
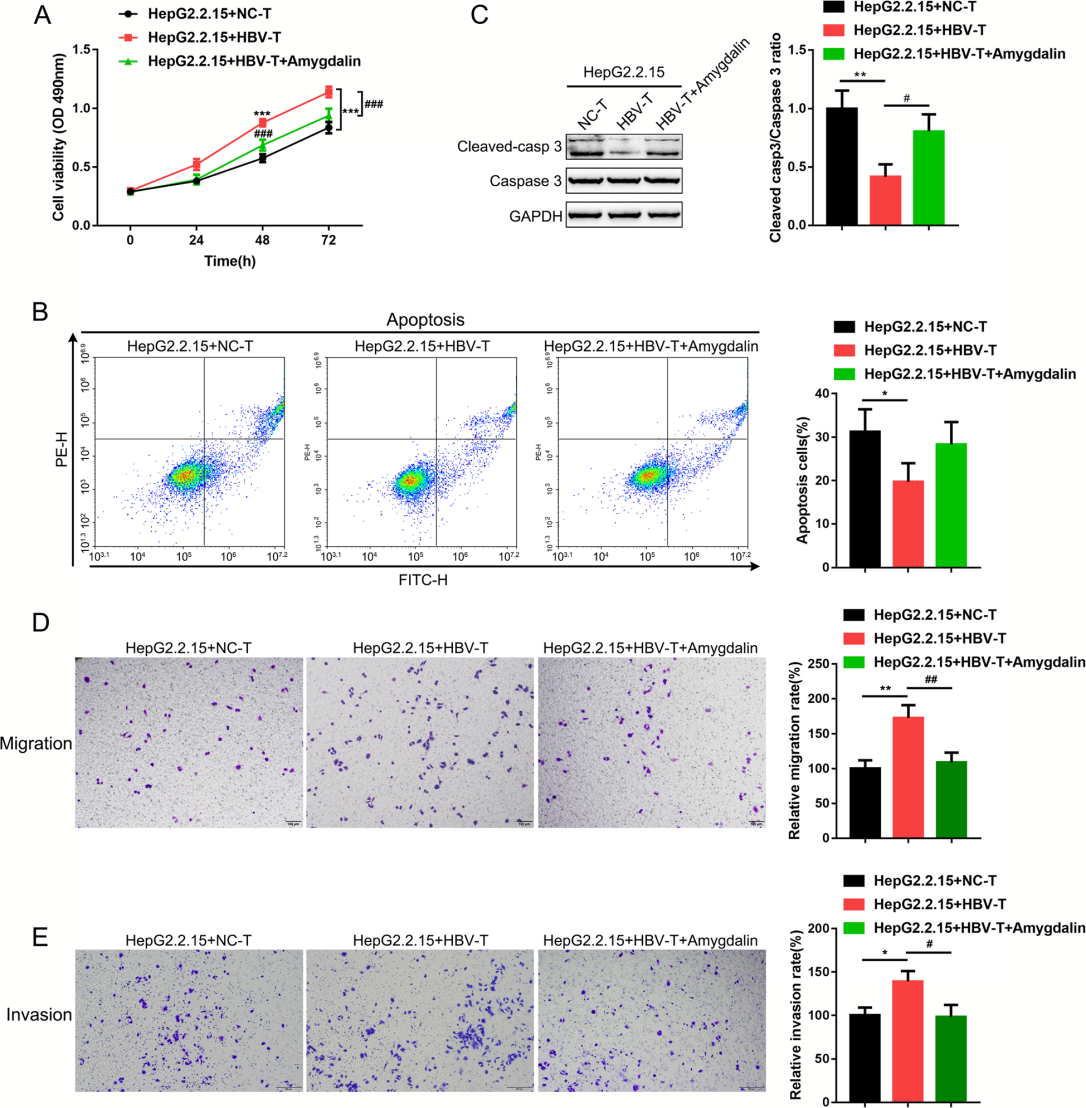
Effects of amygdalin on HepG2.2.15 cell viability, apoptosis, invasion, and migration. **a**-**d** HepG2.2.15 cells were co-cultured with NC-T, HBV-T cells or amygdalin treated HBV-T cells and examined for the cell viability, apoptosis, caspase-3 cleavage, invasion, and migration of HepG2.2.15 cells using MTT (**a**), Flow cytometry (**b**), immunoblotting (**c**), and Transwell assays (**d**, **e**) *n* = 5 for MTT assay, *n* = 3 for flow cytometry, transwell assays and immunoblotting. *p < 0.05, **p < 0.01, compared to HepG2.2.15 + NC-T group, #*p* < 0.05, ##*p* < 0.01, compared to HepG2.2.15 + HBV-T group

As amygdalin affects HBV-T cells to act on cytokine production in HepG2.2.15 cells, next, we investigated whether amygdalin could also affect HepG2.2.15 cell viability, apoptosis, invasion, and migration via HBV-T cells. As shown in Fig. 4a, compared to co-culture with NC-T cells, co-culture with HBV-T cells significantly increased the cell viability of HepG2.2.15 while amygdalin treatment suppressed the cell viability induced by co-culture. Consistently, compared to co-culture with NC-T cells, co-culture with HBV-T cells inhibited HepG2.2.15 cell apoptosis, while amygdalin treated HBV-T cells promoted HepG2.2.15 cell apoptosis (Fig. 4b). Moreover, co-culture with HBV-T cells reduced the expression of cleaved caspase 3 while amygdalin treated HBV-T cells reversed those reduction, further indicating the protive effect of amygdalin on apoptosis (Fig. 4c). Compared to co-culture with NC-T cells, Co-culture with HBV-T cells significantly enhanced the invasion and migration of HepG2.2.15 cells, which could be inhibited by amygdalin therapy (Fig. 4d-e). In summary, amygdalin treatment could affect the cell viability, apoptosis, invasion, and migration of HepG2.2.15 cells through acting on HBV-T cells.

## Discussion

Here, we demonstrate that amygdalin treatment could rescue the HBV-T cell viability and IFN-γ, TNF-α, and IL-2 production. In HBV-T cells, the MFI levels of CD8^+^ are lower than that in NC-T cells and could be reversed by amygdalin treatment. Moreover, the phosphorylation levels of STAT3 and JAK2 are higher in HBV-T cells, compared to those in NC-T cells, and could be reduced by amygdalin treatment. Co-culture with HBV-T cells could reduce IFN-γ and TNF-α production while increase IL-6 and IL-10 production in HepG2.2.15 cells; these alterations could be partially reversed by amygdalin pretreatment. Finally, co-culture with HBV-T cells significantly promoted the cell viability, inhibited the apoptosis, and promoted the invasion and migration of HepG2.2.15 cells, and these alterations could be partially reversed by amygdalin treatment.

Complex T cell immune responses are associated with HBV infection. Studies on mice infected by HBV revealed that CD4+ subtypes were main regulatory factors of the adaptive immune response to HBV, while CD8+ subtypes were key cytokines that mediate the clearance of HBV [29]. In addition, T cells are the primary cell type in tumor immunity; among them, CD4+ subtypes could modulate the anti-tumor immunity via secreting cytokines, while CD8+ subtypes could conduct the secretion of specific inhibitory cytokines, including IFN-γ and TNF-α. CD4+ subtypes, including Th1 and Th2, could not only promote the cytotoxic effect of CTL cells via secreting IL-2, IL-12, IFN-γ, TNF-α, but also stimulate humoral immunity response, promote antibody production and suppress Th1 factors secretion via secreting IL-4, IL-6, IL-8, and IL-10 [30, 31]. In the present study, amygdalin treatment (10, 15, and 20 μg/ml) significantly increases the cell growth of T cells derived from peripheral blood of healthy donors and also rescues the cell growth of T cells derived from patients with HBV-related HCC. Moreover, HBV-T cells secret fewer IFN-γ and TNF-α but more IL-6 and IL-10 than NC-T cells, while amygdalin pretreatment could rescue IFN-γ and TNF-α production and reduce IL-6 and IL-10 production, indicating that amygdalin could play a role in the development of HBV-related HCC through acting on T cell-mediated tumor immunity.

In the tumor-infiltrating immune cells, such as dendritic cells, natural killer cells, and granulocytes, STAT3 phosphorylation is increased. An intrinsic immune-surveillance system can be activated by the suppression of STAT3 activity in hematopoietic cells, thus inhibiting tumor growth and metastasis [32]. p-STAT3 expression in the CD4+ and CD8+ T cells in peripheral blood is higher in patients with active multiple sclerosis than in healthy patients, besides, in several cases of recurrent sclerosis, the p-STAT3 level is closely related to T cell response function [33]. In summary, cellular immune responses, especially CD4+ and CD8+ T cell-mediated immune responses, are essential for the monitoring of malignant tumors and the control of hepatocellular carcinoma development [34]. Herein, the ratio of CD4+ and CD8+/HBV-T cells is remarkably increased or decreased than that in NC-T cells. More importantly, in line with above-mentioned findings, STAT3 and JAK2 phosphorylation levels also considerably increased in HBV-T cells. It has been reported that lipopolysaccharide (LPS)-induced increases in the protein of liver PI3K, AKT, m-TOR, TIMP-1, STAT3, and JAK2 in LPS-treated rats were suppressed by the treatment of 1.5 mg/kg Amygdalin, therefore reducing LPS-induced chronic liver injury in rats by down-regulating the PI3K/AKT, JAK2/STAT3 and NF-κB signaling pathways [35]. Interestingly, we find that amygdalin pretreatment increases HBV-T cell activity and significantly reduces the levels of p-STAT3 and p-JAK2, further indicating that amygdalin could affect T cell activity.

It has been proposed that tumors can increase the production of Th2 cytokines, such as IL-4, IL-6, IL-8, and IL-10. IL-4 and IL-10 are negative regulators in the antitumor immune responses, and they can inhibit IL-2, IL-12, IFN-γ, and TNF-α production by Th1 cells [30]. In the serum of patients with hepatocellular carcinoma and supernatants of peripheral monocytes cocultured with Huh7 cells, the levels of cytokines (IL-4, IL-6, and IL-10) could be upregulated, while the level of IFN-γ could be downregulated [17]. In the present study, co-culture with HBV-T cells also significantly reduced IFN-γ and TNF-α production but increased the secretion of IL-6 and IL-10 in HepG2.2.15 cells, while co-culture-induced alterations in cytokines production in HepG2.2.15 cells could be significantly reversed by amygdalin treatment. More importantly, co-culture with HBV-T cells leads to an increase in the cell viability, invasion, and migration and a decrease in cell apoptosis of HepG2.2.15 cells, while amygdalin treatment significantly reverses the effects of HBV-T cell co-culture on HepG2.2.15 cells.

These findings all indicate that amygdalin treatment could increase the HBV-T cell activity via suppressing the phosphorylation of STAT3 and JAK2, subsequently increases antitumor cytokines production, such as IFN-γ and TNF-α, finally inhibiting the cell growth, invasion, and migration while increasing HepG2.2.15 cell apoptosis. In summary, our in vitro findings provide a rationale for further studies on the functions and mechanism of amygdalin inhibiting HBV-related HCC cell proliferation, invasion, and migration via T cell-mediated tumor immunity.

Regarding the shortcomings of the present study, the present conclusion is based on in vitro experiments; thus, it is unreasonable to draw any conclusion on the in vivo benefits of amygdalin. Considering increasing evidence have been proposed on the in vitro anti-tumor effects of amygdalin while few positive results have been reported based on in vivo studies, in vivo investigation on the role of amygdalin is of great importance. Notably, the risk of serious adverse effects from cyanide poisoning after laetrile or amygdalin, especially after oral ingestion [36, 37], should be considered in in vivo investigation and, more importantly, other drug-delivery ways should be investigated in the meantime.

## Conclusion

Our findings confirmed that amygdalin inhibits HBV-related HCC cell proliferation, invasion, and migration via T cell-mediated tumor immunity.

## Supplementary Information

**Additional file 1: Table S1.** The clinical characteristic.

**Additional file 2: Table S2.** The primer seuqence.

## Data Availability

The datasets used and analysed during the current study are available from the corresponding author on reasonable request.
