# Supplementary material for: Amygdalin promotes the activity of T cells to suppress the progression of HBV-related hepatocellular carcinoma via the JAK2/STAT3 signaling pathway
Source: BMC Infect Dis. 2021 Jan 12;21:56. doi: 10.1186/s12879-020-05713-0 (PMC7802162; doi:10.1186/s12879-020-05713-0)
Supplement: Supplementary file 1 — Additional file 1: Table S1. The clinical characteristic. [file 12879_2020_5713_MOESM1_ESM.docx]

Table S1 the clinical characteristic

| No. | Gender | TNM stage | HBV positive |
| --- | --- | --- | --- |
| N1 | Male | Normal | NO |
| N2 | Male | Normal | NO |
| N3 | Female | Normal | NO |
| N4 | Male | Normal | NO |
| N5 | Female | Normal | NO |
| N6 | Male | Normal | NO |
| N7 | Male | Normal | NO |
| N8 | Female | Normal | NO |
| N9 | Male | Normal | NO |
| N10 | Female | Normal | NO |
| P1 | Male | T2N1M0 | YES |
| P2 | Male | T2N0M0 | YES |
| P3 | Male | T1N0M0 | YES |
| P4 | Female | T1N0M0 | YES |
| P5 | Male | T1N0M0 | YES |
| P6 | Female | T1N0M0 | YES |
| P7 | Male | T2N0M0 | YES |
| P8 | Female | T3N0M0 | YES |
| P9 | Male | T2N1M0 | YES |
| P10 | Male | T1N0M0 | YES |

N：normal volunteers; P: patients
