# Supplementary material for: Amygdalin promotes the activity of T cells to suppress the progression of HBV-related hepatocellular carcinoma via the JAK2/STAT3 signaling pathway
Source: BMC Infect Dis. 2021 Jan 12;21:56. doi: 10.1186/s12879-020-05713-0 (PMC7802162; doi:10.1186/s12879-020-05713-0)
Supplement: Supplementary file 2 — Additional file 2: Table S2. The primer seuqence. [file 12879_2020_5713_MOESM2_ESM.docx]

Table S2 the primer seuqence

| Gene name | Forward | Reverse |
| --- | --- | --- |
| STAT3 | CAGCAGCTTGACACACGGTA | AAACACCAAAGTGGCATGTGA |
| JAK2 | TCTGGGGAGTATGTTGCAGAA | AGACATGGTTGGGTGGATACC |
| GAPDH | ACAGCCTCAAGATCATCAGC | GGTCATGAGTCCTTCCACGAT |
